# Do entrustment scales make a difference in the inter-rater reliability of the workplace-based assessment?

**DOI:** 10.1080/10872981.2022.2053401

**Published:** 2022-03-21

**Authors:** Ayat Nabil Eltayar, Soha Rashed Aref, Hoda Mahmoud Khalifa, Abdullah Said Hammad

**Affiliations:** a Medical Education Department, Faculty of Medicine, Alexandria University, Egypt; b Community Medicine and Public Health Department, Faculty of Medicine, Alexandria University, Egypt; c Histology and Cell biology Department, Faculty of Medicine, Alexandria University, Egypt; d Orthopaedic and Traumatology Department, Faculty of Medicine, Alexandria University, Egypt

**Keywords:** Entrustment scales, performance descriptors, inter-rater reliability, workplace-based assessment

## Abstract

**Background:**

A workplace-based assessment (WBA) is used to assess learners’ competencies in their workplaces. Many workplace assessment tools are available and validated to assess various constructs. The implementation of workplace-based assessment requires proper training of the staff.

**Objective:**

This study aimed to explore the impact of staff training on WBA practices and evaluate the inter-rater reliability of these practices while using entrustment scales, performance descriptors, and personal judgment.

**Design:**

A quasi-experimental study, in which the staff members of the orthopedic department were invited to participate in a training program on the use of entrustment scales and assessment descriptors within the WBA tools. As a response to the training, subjective judgment was replaced by entrustment scales and performance descriptors in a trauma course offered by the orthopedic department. The inter-rater reliability of the WBA was evaluated using various rating scales.

**Results:**

The entrustment scales had higher inter-rater reliability of the assessment tools than performance descriptors and the personal judgment.

**Conclusion:**

The inter-rater reliability was highest when using entrustment scales for WBAs, which could indicate that the entrustment scales achieve good psychometric properties as regards consistency among different raters. Thus, they decrease the confounding effect of differences in assessors. They may also give a clearer image of the actual academic level of the learners.

## Introduction

Workplace-based assessment (WBA) is an authentic assessment approach. It is used to assess the actual performance of a physician in healthcare settings in his/ her everyday work. It has been used for postgraduate assessment since 2005. Recently, it was included in the undergraduate assessment as well. Unlike the traditional clinical assessment methods, WBA is not a snapshot of a learner’s performance and competence in an artificial assessment setting. WBA tools are to be used multiple times during the training to monitor the development and advancement of learners in their actual daily working environment. [1]

Based on the first Miller’s pyramid, the WBA is concerned with assessments at the highest level of the pyramid (Does level). At the ‘Does’ level, the everyday performance of the learner as a physician is assessed. [2]

Various WBA tools are validated for use in assessment purposes in medicine and many specialties. For example, the mini clinical evaluation exercise (mini-CEX) [3] is used to assess history taking, clinical examination, and communication skills as in the clinic or ward. Additionally, the direct observation of procedural skills (DOPS) [3] is used to assess procedural skills as in the trauma center. Moreover, procedure-based assessment (PBA) [3] is used to assess preoperative, operative, and postoperative skills in the operating theater. Besides, a mini peer assessment tool (mini-PAT) [3] is used to assess peers in communication and teamwork.

The first generation of WBA tools used performance descriptors that help the assessors to evaluate the performance of learners based on detailed performance criteria (rubrics). Recently, with the concept of entrusted professional activities, there was a move toward entrustment scales in the WBA tools. An entrustable professional activity is a key task in a training program that a learner can be trusted to perform in each healthcare context when the learner achieves competence. [4]

In addition to validity, reliability is an important aspect of the psychometric properties of any assessment tool. Reliability is concerned with consistency in assessment among raters and over time. [5] The reliability of the WBA tools is improved by increasing the number of assessment points. Additionally, training of the staff may affect the inter-rater reliability and objectivity of any assessment methods. The reliability of any assessment method can be evaluated using Cronbach’s alpha. According to the value of Cronbach alpha, the reliability is classified into five categories. A Cronbach alpha value of ≥ 0.4 denotes a very good level of reliability. A value of 0.3–0.39 denotes a good level of reliability, a value of 0.2–0.29 denotes a marginal level of reliability, and a value less than 0.2 denotes a poor level of reliability.

Evaluations of the training activities help judge their value. [6] There are various models for evaluation. The Kirkpatrick model is one of the commonly used evaluation models. In the Kirkpatrick model, the level of impact of the evaluand is judged. The primary version of the Kirkpatrick pyramid consists of four levels. The lower level is the reaction or satisfaction. At this level, the perception of participants in the training/learning program or workshop is explored. The second level is learning. At this level, any change in the knowledge, skills, or attitude of participants is monitored. In level 3, participants’ behavioral changes are explored in their workplaces. This level needs time and continuous monitoring. The fourth level is concerned with evaluating the results of the training on the performance of the organization. [7]

The scoping review by Chan et al. [8] recommended that more work must be done to provide stronger evidence on both quantitative and qualitative data collected using the WBA. Moreover, the scoping review by Andreson et al. [9] revealed that a limited number of studies discussed the role of subjectivity and bias in WBAs at the level of individual assessors. Andreson et al. [9] recommended further research studying subjectivity while using the WBA tools to fill the gaps in knowledge.

After reviewing the gaps in the literature on the implementation of WBA tools, we aimed to answer the following questions:

What is the impact of staff training on participants? Do the entrustment scales influence the inter-rater reliability of WBA tools?

## Methods

### Study participants

In the first phase, all staff members and teaching assistants in the orthopedic surgery department were invited to participate in an online training program on ‘WBA practices.’

In the second phase, grades given by the assessors of 33 learners enrolled in the hybrid trauma b course in 2020 and 2021 and those of 32 learners enrolled in the traditional face-to-face design in 2018 and 2019 were studied.

### Study design

We carried out a quasi-experimental study in two phases. The first phase was staff training, during which we used Zoom, and there were 16 attendees. We adopted the experiential Kolb’s cycle [10] in our design for the training session. The first step (concrete experience) consisted of exploring the experience of the attendee as regards the clinical assessment. They used to implement a final case-based discussion (CBD) exam in the postgraduate courses. In the second step (reflective observation), they reflected on their previous experience. The experience was considered subjective because they did not use standardized rubrics. In the third step (abstract conceptualization), an interactive lecture about the use of entrustment scales in the WBA in orthopedics was presented. Then, in the fourth step (active experimentation), scenarios tailored to orthopedic surgery were used for training. Those scenarios were adopted from the ‘staff development on WBA’ workshop launched by the Royal College of Physicians and Surgeons in Canada. [6] Then, the scenarios were modified to fit the orthopedic specialty.

### One example of the modified scenarios used in the staff training

A trainee in the surgical theatre is practicing fixation of femur fracture using intramedullary nailing. He is aware of the sterile techniques and how the results will assist in further management. However, he requires direction on patient positioning, land-marking, and the appropriate implant. You would have expected that at his level, he would know and be able to complete these technical portions of the procedure. [6]

Participants were asked to rate the trainee in the scenario using the entrustment scales (Table 1) [11] and share out the score they gave. Assessors were asked to rate training scenarios using performance descriptors. Additionally, they were invited to rate training scenarios using personal judgment without any rubrics. The concept of constructive, descriptive, non-judgmental, and non-personal feedback was presented during the training. Attendees were informed that delivery of the feedback had to be the final step in the assessment using WBA tools. The aim of the feedback was explained as changing ‘*assessment of learning*’ to ‘*assessment for learning*.’ Examples of descriptive and judgmental expressions were explained. For example, if the student attended the clinic late, then the description in the feedback could be ‘*he was late*’ not ‘*he was careless*.’

**Table 1.** Entrustment scale [11]

Level	Descriptor
1	Requires complete guidance; ‘I had to do.’ i.e., The learner requires complete hands-on guidance, ‘did not do’ or ‘was not allowed to do.’
2	Able to perform with repeated direction; ‘I had to talk them through.’ i.e., The learner can perform tasks but requires constant direction.
3	Some independence but intermittent prompting required; ‘I had to direct them from time to time.’ i.e., The learner demonstrates some independence but requires intermittent direction.
4	Independent for most things but requires assistance for nuances; ‘I had to be there just in case.’ i.e., The learner showed independence but was unaware of risks and still requires supervision for safe practice.
5	Complete independence: ‘I did not need to be there.’ i.e., complete independence understands risks and performs safely, practice-ready.

The second phase was implementing what was learned in the workshop. The attendees (staff or orthopedics) were invited to use WBA tools with the entrustment scales to assess their learners in postgraduate courses offered by the Orthopedic and Traumatology Department to provide fairer assessments.

All the attendees were invited to use the WBA tools with the entrustment scales and the performance descriptors within their program of assessment.

The first opportunity to evaluate the level of impact of the training on changing the assessment practices in the orthopedic department was through the trauma B course. It is a 15-week course in the orthopedic master’s degree program. It exposes learners to common concepts in the management of trauma patients. It includes the following topics: Biomechanics of Fractures, Pathological and Stress Fractures, Peri-prosthetic Fractures, Osteoporotic Fractures, Injuries in Children, Compartmental Syndrome, Implant Failure, Poly-trauma Patients, Dislocations, Injuries in the Elderly, and Sports Injuries.

The trauma B course was delivered in a traditional lecture-based, face-to-face clinical training format. The assessment was in the form of an end-of-course multiple choice question (MCQ) exam and a CBD by two assessors.

In 2020, the course was changed to a hybrid format to provide more flexible distant lectures while maintaining the clinical training in a face-to-face format. A program of assessment was created for the hybrid course. It included continuous assessments in the form of weekly low-stake online assignments such as gaming, problem- and script-based activities, quizzes, and script-concordance tests. After the staff training, WBAs were used eight times all through the course. The high-stake assessment was in the form of MCQs, modified essay questions, and CBD by the end of the course. The written exam was built based on the test blueprint created for the course; however, this is out of the scope of evaluation in this manuscript.

The used WBA tools were the mini clinical evaluation exercise (mini-CEX), DOPS, procedure-based assessment (PBA), mini-PAT, and CBD. Each of the previously mentioned tools was implemented twice during the course, by a single assessor each time, except for the CBD. The CBD was implemented only once during the course, by two assessors. Entrustment scales (Table 1) were used in the mini-CEX, DOPS, and PBA. [9] However, in the CBD, performance descriptors (Table 2) were used. Feedback was recorded by the staff and self-evaluation was recorded by the students.

**Table 2.** Performance descriptors

Rating	Descriptor
0	Demonstrates little knowledge and lacks the ability to evaluate issues resulting in only a rudimentary contribution to the management plan
1	Demonstrates some knowledge and limited evaluation of issues resulting in a limited management plan
2	Demonstrates satisfactory knowledge and logical evaluation of issues resulting in an acceptable management plan consistent with higher training

The study aims to evaluate the inter-rater reliability for the different practices in the WBA. The staff training was also evaluated reflecting on the Kirkpatrick evaluation model. [12]

### Data collection methods and tools

In phase 1, group discussions with the attendees of the staff training activity were used to evaluate the training activity. Discussions that lasted for 15–20 minutes were held online by the end of the training. The attendees were invited to participate, and their comments were recorded.

In phase 2, learners’ grades reported by different assessors while using WBA tools were used to calculate the inter-rater reliability.

### Outcome measures

- Assessing the impact of the staff training on the participants.
- Evaluation of the inter-rater reliability of the WBA tools using entrustment scales, performance descriptors (after the training), and experts’ judgment (without training).

### Data processing and analysis

For qualitative data, the descriptive and interpretive approach for data analysis was adopted. Note-based analysis and memory-based analysis were used for transcription.

Quantitative data were coded, checked for errors, and entered into Statistical Package for the Social Sciences Version 22.0 software for statistical analysis. Data were summarized and presented using the appropriate statistics. Descriptive statistics: Frequencies, percentages, means, standard deviations and medians were used to present the data. Quantitative variables were tested for normality using the Shapiro-Wilk test.

## Results

In phase 1, a thematic analysis was carried out to analyze qualitative data. The following themes emerged from the participants’ discussion on their assessment practices and the impact of the staff training.

### Criticizing the past experience

By the end of the staff training, attendees were asked to describe their past practices in assessment. The emerged subthemes were personal diligence and accuracy. Many of them described the assessment practices as personal diligence. They declared that the assessment of learners was based on the personal judgment of the assessors without using any rubrics, and without undergoing training in the assessment practices.

Some of them agreed that a single assessment point can’t give accurate significant data on the competency level of the learner.

### Improving future practices

During the staff training, there was a consensus on the grades given by the trainees while using the entrustment scales as compared to those given while using the performance descriptors and personal judgment. Then, during discussions, the subthemes of feasibility and training time were raised. Most staff members confirmed that the entrustment scales were used with no need for creating a frame of reference for the criteria of performance in each clinical skill. For example, one of the trainees mentioned that:

The entrustment scales were simple to use and uniformly interpreted by the assessors.

Many of them considered that they required little training time to master the assessment using the entrustment scales.

### Expected challenges for future assessment plans

Time constraints and record storage were the main subthemes. Some of the staff members expressed their worries about the time constrains for them as clinicians to record written feedback, which they considered a demanding task. Most of them considered keeping these records of learners’ performances challenging. For example, one of the staff said:

The documentation of feedback and the continuous assessment are demanding. We are overwhelmed with our duties in patient care.

After exploring the satisfaction and perception of participants by the end of the staff training, its impact at level 4 of the Kirkpatric model was evaluated. At this level, the focus of the evaluation was on the results (did the change in the assessment practices of the staff after the training have a positive impact?). The previously asked question will be answered in phase 2 when evaluating the inter-rater reliability in different WBA assessment practices

In phase 2, Table 3 describes the distribution of learners’ grades in percentages in different points of assessment. There were 33 learners in PBA, DOPS, and mini-CEX. Similarly, there were 33 learners in CBD with performance descriptors. However, there were 32 learners in CBD without rubrics.

**Table 3.** Learners’ grades using different workplace-based assessment (WBA) tools

Assessment (n. of assessment point)	Min%–Max%	Mean of percent scores ± SD	
PBA (1)	50.10–90	69.92 ± 10.20	
PBA (2)	55–95	73.14 ± 9.94	
DOPS (1)	52–91	70.11 ± 10.44	
DOPS (2)	53–95	73.27 ± 9.90	
mini-CEX (1)	50–92	70.26 ± 10.04	
mini-CEX (2)	52–96	73.68 ± 9.61	
CBD (1)	45–84	57.97 ± 12.59	
CBD (2)	46–95	83.51 ± 11.99	
	Min% – Max%	Mean of percent scores ± SD	** Median of percent scores
CBD* (1)	15–87	68.66** ± **17.76	57
CBD* (2)	26–99	84.72** ± **17.10	69.5

*CBD grades of students without using any rating rubrics (before undergoing the staff training)

**CBD data were not normally distributed.

Moreover, Table 3 shows the grades of learners in PBA, DOPS, and mini-CEX using the entrustment scales and the CBD, in which the performance description scales were used. However, the CBD* describes the learners’ grades in percentages (without using any rating rubrics, before undergoing the training).

On correlating the grades of students in the two points of assessment of PBA (PBA 1 and PBA 2), there was a strong significant association (Spearman’s correlation coefficient of 0.98, p ˂ 0.001). Regarding DOPS 1 and 2, there was a strong significant association (Spearman’s correlation coefficient of 0.95, p ˂ 0.001). Concerning mini-CEX 1 and 2, the correlation was strongly positive as well (Spearman’s correlation coefficient of 0.96, p ˂ 0.001). On correlating the grades of the CBD rated by two different assessors for each student using the performance descriptors, there was a significant strong association (Spearman’s correlation coefficient of 0.81, p ˂ 0.001). Regarding the CBD, rated by two assessors holistically and based on personal judgment, the association was moderate and positive (Pearson’s correlation coefficient of 0.40, p ˂ 0.001).

The reliability of each assessment was calculated using the Cronbach alpha. The Cronbach alpha of the two assessment points of mini-CEX was 0.81 while that of DOPS was 0.74. Additionally, the Cronbach alpha of the two assessment points of the PBA was 0.68. However, the Cronbach alpha of the ratings of the two assessors in the CBD while using the performance descriptors was 0.54. All the previously mentioned assessment points were implemented after undergoing the staff training. The Cronbach alpha of the ratings of the two assessors in the CBD exam while using personal judgment (before the staff training) was 0.27.

## Discussion

Our findings revealed that staff training contributed to changing the behavior of the staff as regards using the entrustment scales WBA tools, which in turn improved the inter-rater reliability of assessment as compared to those using performance descriptors and those depending on subjective judgment. Additionally, the grades of the two different assessors in the WBA tools showed that using the entrustment scales had better associations between the grades given by the two assessors. This can be explained by the consistency of the entrustment scales that is more than that of the performance descriptors and personal judgment. During the staff training, it was clear that there was a consensus in the grades given by the assessors (trainees) using the entrustment scales. However, there was a discrepancy in the grades given by some of the staff while using the performance descriptors. The reason for this could be the different interpretations of the performance descriptors. On the contrary, the personal judgment without using a guiding rubric revealed more discrepancies between the assessors. This could be explained by the difference in the level of expertise among the assessors.

Trainees mentioned that they did not need lengthy training to master the use of the entrustment scales. They might have perceived this as the performance descriptors needing a stage of discussion and a consensus on the standards of performance for each clinical competency.

On exploring similar studies, the one by Holzhausen et al. [13] explored the use of entrustment scales in an objective structured clinical exam (OSCE) and revealed that most of the assessors positively evaluated the addition of entrustment rating scales into the OSCE. Additionally, Rekman et al. [14] explained that the entrustment scales had great potential for synchronizing the clinical assessors’ judgment with the competency-rating scale.

There is a paucity of available studies on the evaluation of the inter-rater reliability of WBA tools using different rating strategies. According to the findings of this study, the inter-rater reliability of the WBA using entrustment scales was higher than that of those using performance descriptors and those depending on subjective judgment.

## Limitations

This study had some limitations. The study design we chose may not have been the best for this kind of study. A randomized controlled experimental design would have stronger evidence than the quasi-experimental design we used. Additionally, the number of participants in the staff training activity was limited because the study was carried out at a single clinical department as a first step. Future studies with larger samples involving multiple clinical departments could provide stronger evidence.

## Conclusion

By the end of the staff training, most of the participants showed positive reactions toward the use of entrustment scales within their WBA tools. Many agreed that they needed little time to master the use of the entrustment scales. They expressed their worries about the time constraints in providing written feedback. The majority of them considered the storage of WBA data challenging. In the second phase, the evaluation of the impact of the staff- training on the assessment practices using the Kirkpatrick evaluation model, the staff members were encouraged to implement what they had learned in the training about the use of entrustment scales in WBA tools. Finally, the WBA using the entrustment scales showed better inter-rater reliability than those using performance descriptors and those depending on subjective judgment.

## References

[1] Yousuf Guraya S. Workplace-based assessment; applications and educational impact. Malays J Med Sci. 2015;22(6):5–6. PMC5295751 28223879

[2] Miller GE. The assessment of clinical skills/competence/performance. Acad Med. 1990;65(9):S63–7. doi:10.1097/0000188-199009000-00045. 2400509

[3] Norcini J, Burch V. Workplace-based assessment as an educational tool: AMEE Guide no. 31. Med Teach. 2007;29(9–10):855–871. 18158655 10.1080/01421590701775453

[4] Ten Cate O. Trust, competence, and the supervisor’s role in postgraduate training. BMJ. 2006;333(7571):748–751. 17023469 10.1136/bmj.38938.407569.94 PMC1592396

[5] Haneline M, Cooperstein R. Weighing the reliability and validity of clinical tests. J Chiropr Med. 2006;43:19–22.

[6] Dudek N, Gofton W, Bhanji F. Workplace-based assessment practical implications [presentation] Ottawa: the Royal College of Physicians and Surgeons Canada 2017. [cited 2021 Oct]. Available from:: http://www.royalcollege.ca/rcsite/documents/cbd/work-based-assessment-practical-implications-program-directors-e.pptx

[7] Frye ANNW, Hemmer PA. Program evaluation models and related theories: AMEE Guide no. 67. Med Teach. 2012;67(5):288–299. 10.3109/0142159X.2012.668637 22515309

[8] Chan TM, Sebok-Syer SS, Cheung WJ, et al. Workplace-based assessment data in emergency medicine: a scoping review of the literature. AEM Educ Train. 2021;5. 10.1002/aet2.10544 PMC8166307 34099992

[9] Anderson HL, Kurtz J, West DC. Implementation and use of workplace-based assessment in clinical learning environments: a scoping review. Acad Med. 2021;96:S164–74. 34406132 10.1097/ACM.0000000000004366

[10] Russ V. Behind and beyond Kolb’s learning cycle. J Manag Edu. 1998;22:304–319.

[11] Gofton WT, Dudek NL, Wood TJ, et al. The Ottawa surgical competency operating room evaluation (O-SCORE): a tool to assess surgical competence. Acad Med. 2012;87(10):1401–1407. 22914526 10.1097/ACM.0b013e3182677805

[12] Shih-Chieh L, Shih-Yun H. Evaluating a continuing medical education program: new world Kirkpatrick model approach. Ijmess. 2019;8:266–279.

[13] Holzhausen Y, Maaz A, März M, et al. Exploring the introduction of entrustment rating scales in an existing objective structured clinical examination. BMC Med Educ. 2019;19(1):319. 31438938 10.1186/s12909-019-1736-2 PMC6704513

[14] Rekman J, Gofton W, Dudek N, et al. Entrustability scales: outlining their usefulness for competency-based clinical assessment. Acad Med. 2016;91(2):186–190. 26630609 10.1097/ACM.0000000000001045

